# Reliability of an all-in-one wearable sensor for continuous vital signs monitoring in high-risk patients: the NIGHTINGALE clinical validation study

**DOI:** 10.1007/s10877-025-01279-x

**Published:** 2025-03-18

**Authors:** Martine J. M. Breteler, Ellen Leigard, Lisa C. Hartung, John R. Welch, David A. Brealey, Sebastian J. Fritsch, David Konrad, Daniel Hertzberg, Max Bell, Heleen Rienstra, Frank E. Rademakers, Cor J. Kalkman

**Affiliations:** 1 Department of Anesthesiology, University Medical Center Utrecht, Utrecht University, Utrecht, The Netherlands; 2 Department of Perioperative Medicine and Intensive Care, Karolinska University Hospital, Stockholm, Sweden; 3 Department of Physiology and Pharmacology, Karolinska Institutet, Stockholm, Sweden; 4 Department of Intensive Care Medicine, University Hospital RWTH Aachen, Aachen, Germany; 5 Division of Critical Care, University College London Hospitals NHS Foundation Trust, London, UK; 6 The NIHR University College London Hospitals Biomedical Research Centre, London, UK; 7 NIHR Central London Patient Safety Research Collaboration, London, UK; 8 University Hospitals Leuven, Faculty of Medicine, Leuven, Belgium; 9 Jülich Supercomputing Centre, Forschungszentrum Jülich GmbH, Jülich, Germany; 10 University Medical Center Utrecht, Mailstop Q.04.2.313, P.O. Box 85500, Utrecht, 3508 GA The Netherlands

**Keywords:** Continuous monitoring, Vital signs, Remote monitoring, Clinical deterioration, Wearable device

## Abstract

Continuous vital signs monitoring with wearable systems may improve early recognition of patient deterioration on hospital wards. The objective of this study was to determine whether the wearable Checkpoint Cardio’s CPC12S, can accurately measure heart rate (HR), respiratory rate (RR), oxygen saturation (SpO2), blood pressure (BP) and temperature continuously. In an observational multicenter method comparison study of 70 high-risk surgical patients admitted to high-dependency wards; HR, RR, SpO2, BP and temperature were simultaneously measured with the CPC12S system and with ICU-grade monitoring systems in four European hospitals. Outcome measures were bias and 95% limits of agreement (LoA). Clinical accuracy was assessed with Clarke Error Grid analyses for HR and RR. A total of 3,212 h of vital signs data (on average 26 h per patient) were analyzed. For HR, bias (95% LoA) of the pooled analysis was 0.0 (-3.5 to 3.4), for RR 1.5 (-3.7 to 7.5) and for SpO2 0.4 (-3.1 to 4.0). The CPC12S system overestimated BP, with a bias of 8.9 and wide LoA (-23.3 to 41.2). Temperature was underestimated with a bias of -0.6 and LoA of -1.7 to 0.6. Clarke Error Grid analyses showed that adequate treatment decisions regarding changes in HR and RR would have been made in 99.2% and 92.0% of cases respectively. The CPC12S system showed high accuracy for measurements of HR. The accuracy of RR, SpO2 were slightly overestimated and core temperature underestimated, with LoA outside the predefined clinical acceptable range. The accuracy of BP was unacceptably low.

**Supplementary Information:**

The online version contains supplementary material available at 10.1007/s10877-025-01279-x.

## Introduction

Hospitalized patients can die because early signs of deterioration are missed [1]. Adverse events and complications are usually preceded by abnormal vital signs [2–6] providing opportunities for earlier recognition and timely intervention. Recent studies show that more than 80% of hyoxemic and hypotensive events are missed by intermittent monitoring [7–9], routinely performed once every four to eight hours in hospitalized patients worldwide. Furthermore, early warning scores (EWS) are often incomplete [10–12] or not recorded at all, indicating an unfilled need for better monitoring of vital signs on general hospital wards to improve patient outcomes [13, 14].

Over the past decade, many wireless wearable continuous monitoring solutions have emerged, specifically designed for ‘low-care’ environments. Continuous monitoring systems in combination with predictive models have been reported to facilitate automated recognition of clinical deterioration, diminish the need for Intensive Care Unit (ICU) transfer [11, 12, 15], reduce length of hospital stay and improve survival [14]. However, most wireless vital signs monitors are only capable of measuring a subset of vital signs, often limited to heart rate (HR), respiratory rate (RR) and temperature. Time-consuming intermittent manual measurements of blood pressure (BP) and oxygen saturation (SpO2) are therefore still needed, limiting introduction of such systems in clinical practice [16].

An ‘all-in-one’ wearable patient monitoring solution capable of retrieving a full set of vital signs has recently been developed in the H2020 competitive ‘Nightingale’ Pre-Commercial Procurement (PCP) program funded by the European Commission [17]. Within this EU-funded initiative, five European academic hospitals (Utrecht, the Netherlands; Stockholm, Sweden; London, United Kingdom; Leuven, Belgium and Aachen, Germany) stimulated industry to develop the next generation of wireless wearable sensors for continuous vital signs monitoring in clinical practice. This Nightingale PCP program was funded since no state of the art wireless solutions were on the market that could measure a full set of vital signs (including BP and SpO2) continuously both for use on regular wards as well as at home. Within a competitive scheme, four companies reached the stage to develop a prototype sensor system, during which reliability and usability were tested and validated in healthy volunteers [18]. One of these companies was able to further improve the wearable wireless sensor system which is evaluated in the present study. However, before proceeding to large multicenter interventional trials studying outcomes, it is crucial to validate vital sign measurement performance in real clinical practice [19]. Such clinical validation studies have been rare and robust evidence is lacking, but clinical validation is now required under the European Medical Device Regulation (MDR) 2017/745 since May 26, 2021 [20].

The objective of this study was therefore to evaluate whether this new wearable multi-parameter sensor could accurately measure HR, RR, SpO2, BP and temperature continuously in high-risk patients compared to four *different* standard monitoring systems in four European hospitals. Our secondary aim was to assess clinical accuracy of measurement performance and assess potential consequences for clinical decision making.

## Materials and methods

### Study design

We conducted a multicenter clinical observational study between November 2020 and October 2021 in which 125 high-risk surgical and medical patients were asked to wear the multiparameter ambulatory telemonitoring system CPC12S system ([Checkpoint Cardio Ltd, Kazanlak, Bulgaria]) in-hospital and at home after discharge. Of these patients, 70 could be included in the present method comparison study since they were simultaneously monitored with the CPC12S system and standard bedside monitoring systems in the ICU, High-Dependency Unit (HDU) or Post Anesthesia Care Unit (PACU). The study was conducted in four large academic hospitals: University Medical Center Utrecht, the Netherlands; Karolinska University Hospital, Sweden; University Hospital RWTH Aachen, Germany and Leuven University Hospital, Belgium. The study protocol differed slightly between the centers due to organizational and legislative differences. Therefore, there was variation between hospitals regarding number of study patients, observation time available for agreement analysis and sampling rate of vital signs from the reference systems. HR, RR, SpO2, BP and temperature were continuously monitored with both the CPC12S system and standard reference monitoring systems. To ensure routine hospital care, treating clinicians did not have access to measurement data from the device during the study, and study personnel only accessed the system to verify whether data was transferred. Formal ethical approval of the study was obtained from each of the Medical Research Ethics Committees in Utrecht (No. 20/078), Stockholm (No. 2020–04537), Aachen (No. EK 417/20) and Leuven (No. B3222020000163).

### Study population and setting

Adult patients (≥ 18 years of age) scheduled to undergo major non-cardiac surgery or admitted for an acute medical condition were eligible for inclusion. These patients were considered for enrollment because they belong to a high-risk group more likely to experience deterioration events, with abnormal vital parameters, compared to other patients on general wards. Exclusion criteria were patients with pacemaker or implantable cardioverter defibrillator, allergy to skin adhesives, wounds near the application site or inability to provide informed consent. All patients provided written informed consent before enrollment. The CPC12S system was applied, and vital sign recording started postoperatively after admission to the ICU, HDU or PACU.

### Description of the CPC12S system

**Fig. 1:**
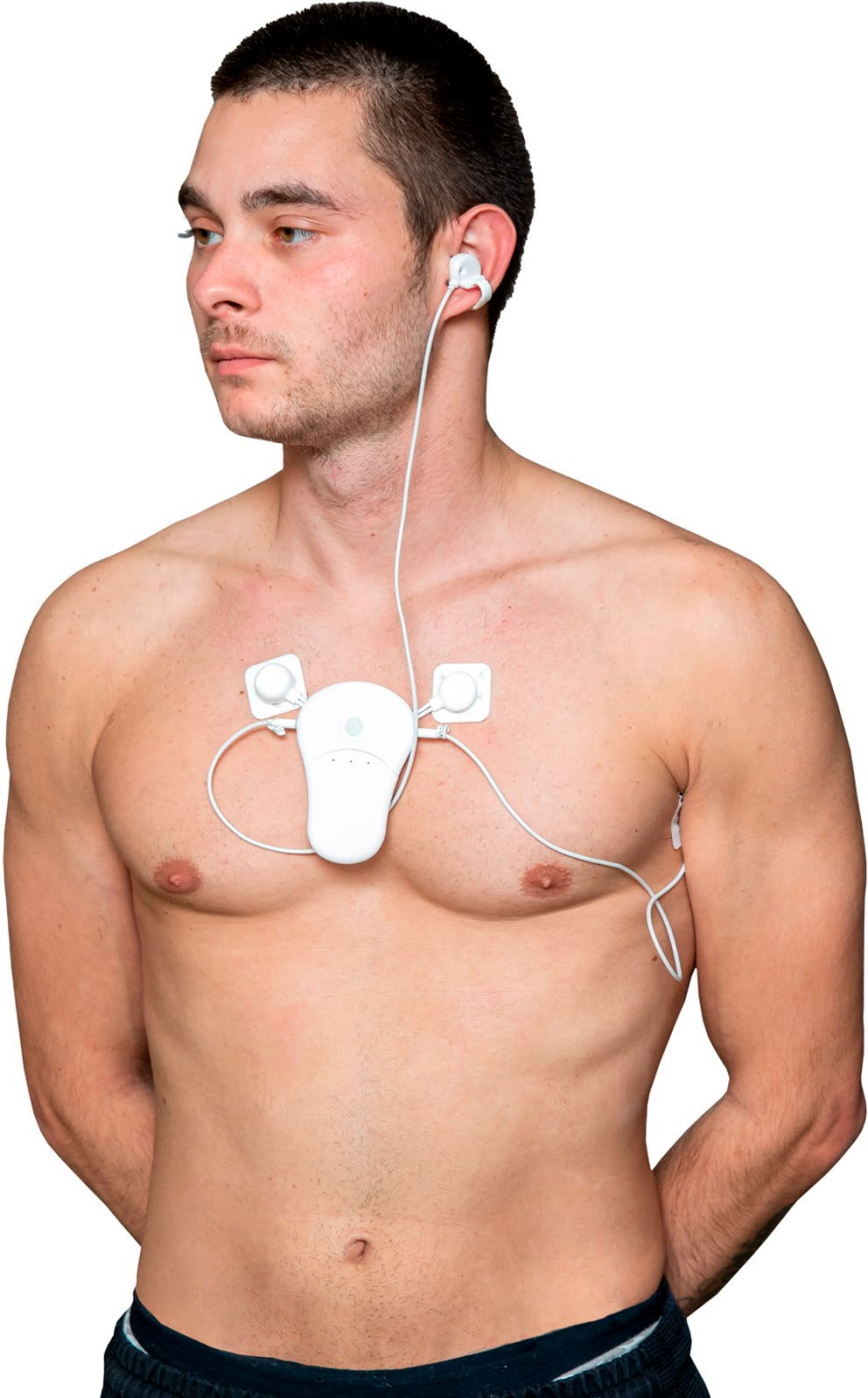
The CPC12S Nightingale multiparameter monitoring system (Checkpoint Cardio Ltd, Bulgaria). The wearable sensor attached with two electrodes on the chest measures ECG and HR. RR is derived using impedance pneumography. The ear sensor measures photoplethysmogram to determine SpO2. BP is derived from pulse transit time (PTT) using signals from both PPG and ECG. Temperature is measured by a thermistor placed in the axilla

The CPC12S system is a reusable, lightweight (90 g) wearable sensor worn on the chest using electrodes measuring electrocardiography (ECG), HR and RR. Both an ear sensor measuring photoplethysmogram (PPG) for determination of SpO2 and BP, and a temperature sensor are connected to the chest-sensor (Fig. 1). Either a single- (Leuven, Stockholm and Utrecht) or three-lead ECG (Aachen) were used. Body position and movement are also registered by the sensor but not evaluated in this study.

The sensor calculates HR by analyzing R-peaks of QRS-complexes in the raw ECG data. RR is recorded using impedance pneumography. SpO2 is determined by analyzing the PPG waveform. BP is derived by calculating the pulse transit time (PTT) using R-peaks from the QRS complexes, peaks in the PPG pulse waves, and timing of the second heart-tone corresponding to the dicrotic notch, measured by a stethoscope in the device [21, 22]. Axillary temperature is measured by a separate thermistor placed in the axilla. All waveforms (ECG, PPG, respiration and stethoscope signal) are saved by the sensor system. The sensor algorithms reject calculation of vital signs if waveform signals are invalid due to contamination by noise. Update frequency of the individual vital signs is every 20 s. Measurements are transmitted via Bluetooth Low Energy (BLE) to an Android cell phone (Blackview A60 pro model [Blackview, Hongkong, China]), that is uploading the data via mobile internet or WiFi to a secured server.

### Description of the reference monitoring systems

The following bedside monitoring systems were used as reference systems: XPREZZON [Spacelabs Healthcare, United States] used at UMC Utrecht, Intellivue MP50 [Philips, the Netherlands]) used by University Hospital RWTH Aachen, Intellivue Mx800 [Philips, the Netherlands] used at both Karolinska University Hospital and Leuven University Hospital. All reference systems used ECG for HR monitoring and measured RR by thoracic impedance pneumography or capnography. BP was measured invasively by an intra-arterial catheter and SpO2 by pulse oximetry. Continuous core temperature was derived from urinary bladder monitoring, and therefore only analyzed in patients with a temperature catheter present. Vital signs data from each reference system was stored every 15–60 s, except for the reference system in Aachen that saved and transmitted one measurement every 15 min.

### Signal analysis

Data from the CPC12S and reference systems were retrieved in comma-separated (CSV) format and processed using MATLAB (The MathWorks, United States). Non-physiological outliers from all systems were removed: HR > 250 beats per minute (bpm), RR > 60 breaths per min (brpm) and SpO2 < 50%. We used mean arterial pressure (MAP) values to analyze BP and removed MAP values > 180 mmHg. Additionally, temperature readings < 34 °C and > 42 °C were removed since frequent and short periods of hypothermia < 34 °C followed by immediate return to normothermia is physiologically impossible, and likely caused by sensor displacement. Data from the CPC12S was averaged to once per minute (i.e., median over 60 s) and compared to the nearest time point forward in time of each reference system. Data from the reference system used at Karolinska University Hospital (transmitted once every 15 s) was averaged to produce paired data with CPC12S every 60 s. Furthermore, sensor and reference data were synchronized to ensure alignment of both time series. After synchronization, a ‘moving’ median filter with a window of 15 min was applied to eliminate movement artifacts.

### Outcomes and statistical analysis

The primary outcome was bias and precision with 95% limits of agreement (LoA) between vital signs measured by the CPC12S system and the reference standards. We considered HR and RR acceptable for clinical purposes if within ± 10% of the reference standard or ± 5 bpm or ± 3 brpm (whichever is greater). For SpO2, BP and temperature we considered the measurements acceptable if within ± 2%, ± 10 mmHg or ± 1 °C respectively [23–25]. All data pairs derived from the CPC12S system and each of the reference standards were analyzed using the Bland-Altman method for repeated measurements [26]. The mean difference (bias) between the CPC12S system and reference standards, and the 95% LoA (± 196 SD) were determined for each of the vital signs after testing whether the differences were normally distributed. In addition, LoA was calculated with a mixed effects model (MEM) using a modification for handling repeated measurements [27, 28]. The MEM involves time as a random effect and adjusts for baseline, average value of each patient over time and the mean measurement between the CPC12S system and each of the reference standards for each measurement. Furthermore, pooled analyses were executed to provide combined estimates of bias and 95% LoA [29].

As secondary outcomes, Clarke Error Grid analyses were used to evaluate clinical accuracy of measurement performance and assess potential consequences for clinical decision making [30]. A Clarke Error Grid represents a scatterplot designed to evaluate the difference between a new method and a reference standard with values assigned to zone A to E. Zone A shows measurements within 20% o the reference monitor; zone B contains measurements outside 20% o the reference, but not leading to unnecessary treatment. Region C contains measurements leading to unnecessary treatment, region D indicates a potentially dangerous failure to detect bradycardia/bradypnoea or tachycardia/tachypnoea, and region E represents points where events are confused (e.g., bradycardia with tachycardia). Clarke Error Grid analyses were conducted for HR and RR [26]. Measurements of BP, SpO2 and temperature are shown in scatterplots to visualize their accuracy.

## Results

From the 70 patients, 3,212 h of vital signs monitoring were available for methods comparison analyses, with a median duration of 26 h (range 3 to 231 h) per patient. Patient characteristics are summarized in Table 1. Total duration of monitoring and average amount of monitored time differed between centers (Table 1). Table 2 shows bias and precision (95% LoA) of comparisons between the CPC12S system and each of the reference standards.

**Table 1:** Patient characteristics (*n* = 70)

	Utrecht (*n* = 18)	Stockholm (*n* = 25)	Aachen (*n* = 24)	Leuven (*n* = 3)	All patients (*n* = 70)
Age in years, median [IQR]	68 [6]	74 [7]	62 [13]	58 [9]	69 [15]
Women, n (%)	6 (33)	17 (68)	7 (29)	2 (67)	32 (46)
Surgical indication, n (%)					
Major upper GI^b^ oncological surgery	18 (100)	0 (0)	19 (79)	1 (33)	38 (54)
Major lower GI^b^ oncological surgery	0 (0)	23 (92)	1 (4)	0 (0)	24 (34)
Other major surgeries (e.g., vascular surgery)	0 (0)	2 (8)	4 (17)	2 (67)	8 (11)
Comorbidities, n (%)					
Lung disease (COPD^a^ or asthma)	2 (11)	7 (28)	0 (0)	0 (0)	10 (14)
Ischaemic heart disease	2 (11)	6 (24)	2 (8)	0 (0)	10 (14)
Heart failure (including valvular diseases)	1 (6)	4 (16)	0 (0)	0 (0)	5 (7)
Atrial fibrillation	1 (6)	3 (12)	1 (4)	0 (0)	5 (7)
Hypertension	7 (39)	18 (72)	11 (44)	1 (33)	37 (53)
Chronic kidney disease	2 (11)	4 (16)	1 (4)	0 (0)	7 (10)
Length of hospital stay, days median [IQR]	11 [10]	7.5 [3]	17 [22]	8 [14]	11 [12]
Total duration of ‘double’ monitoring, hours	936	715	1374	187	3212
Average duration of ‘double’ monitoring in hours, median [IQR]	39 [49]	16 [12]	40 [86]	70 [84]	26 [53]

^a^*COPD*: Chronic obstructive pulmonary disease

^b^*GI*: Gastrointestinal

^c^*IQR*: Inter Quartile Range

### Heart rate

**Fig. 2:**
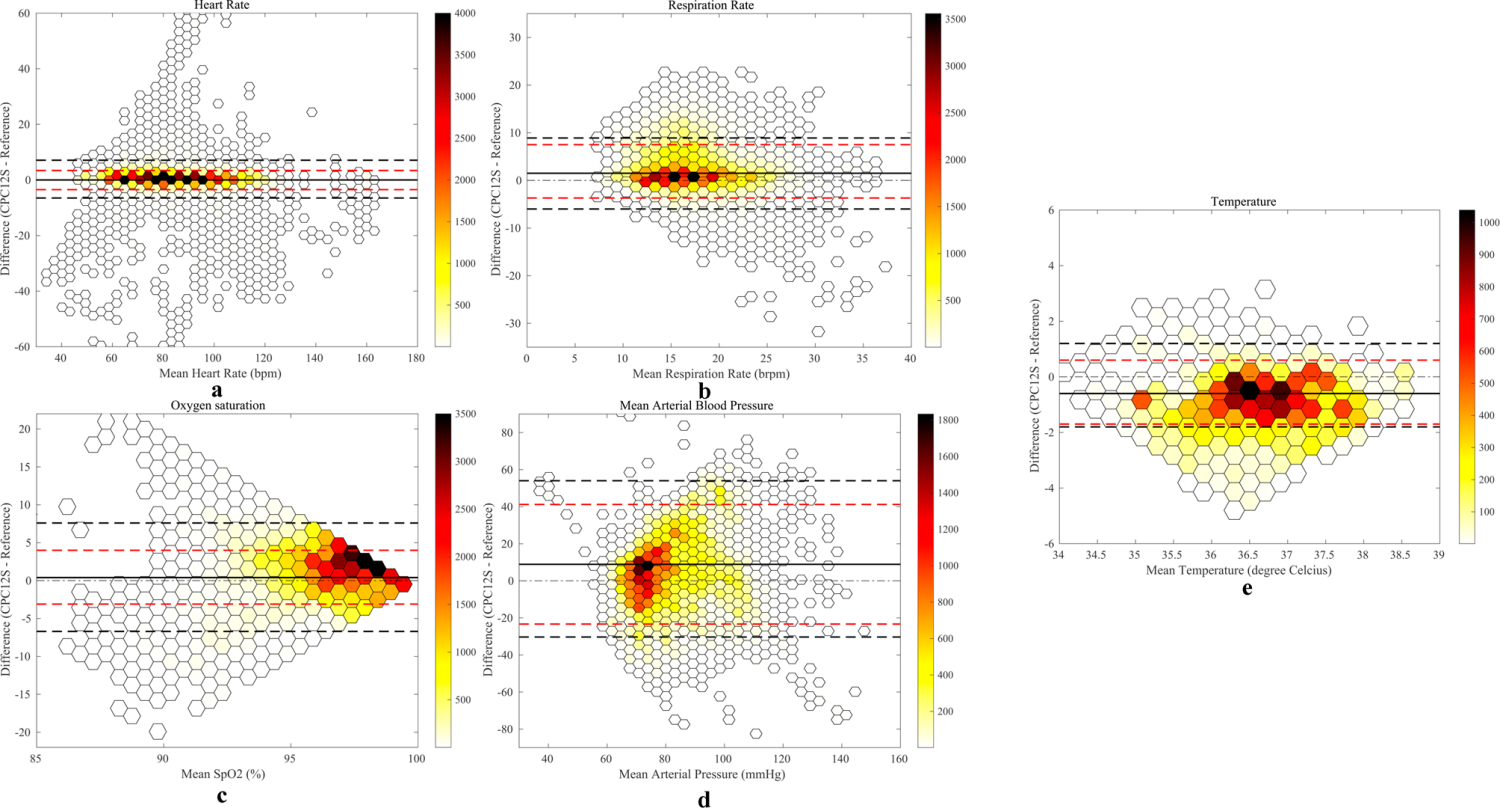
**(a)** Bland-Altman plot of the pooled analysis of all heart rate measurements with few (white) to many (dark red) measurement pairs. The dashed black line corresponds to the limits of agreement from the Bland-Altman method, and the dashed red line from mixed effects models respectively. Bias is shown as a black line. **(b)** Bland-Altman plot of the pooled analysis of all respiratory rate measurements with few (white) to many (dark red) measurement pairs. The dashed black line corresponds to the limits of agreement from the Bland-Altman method, and the dashed red line from mixed effects models respectively. Bias is shown as a black line. (**c**) Bland-Altman plot of the pooled analysis of all oxygen saturation (SpO2) measurements with few (white) to many (dark red) measurement pairs. The dashed black line corresponds to the limits of agreement from the Bland-Altman method, and the dashed red line from mixed effects models respectively. Bias is shown as black line. **(d)** Bland-Altman plot of the pooled analysis of all mean arterial pressure (MAP) measurements with few (white) to many (dark red) measurement pairs. The dashed black line corresponds to the limits of agreement from the Bland-Altman method, and the dashed red line from mixed effects models respectively. Bias is shown as a black line. **(e)** Bland-Altman plot of the pooled analysis of all temperature measurements with few (white) to many (dark red) measurement pairs. The dashed black line corresponds to the limits of agreement from the Bland-Altman method, and the dashed red line from mixed effects models respectively. Bias is shown as a black line

**Fig. 3:**
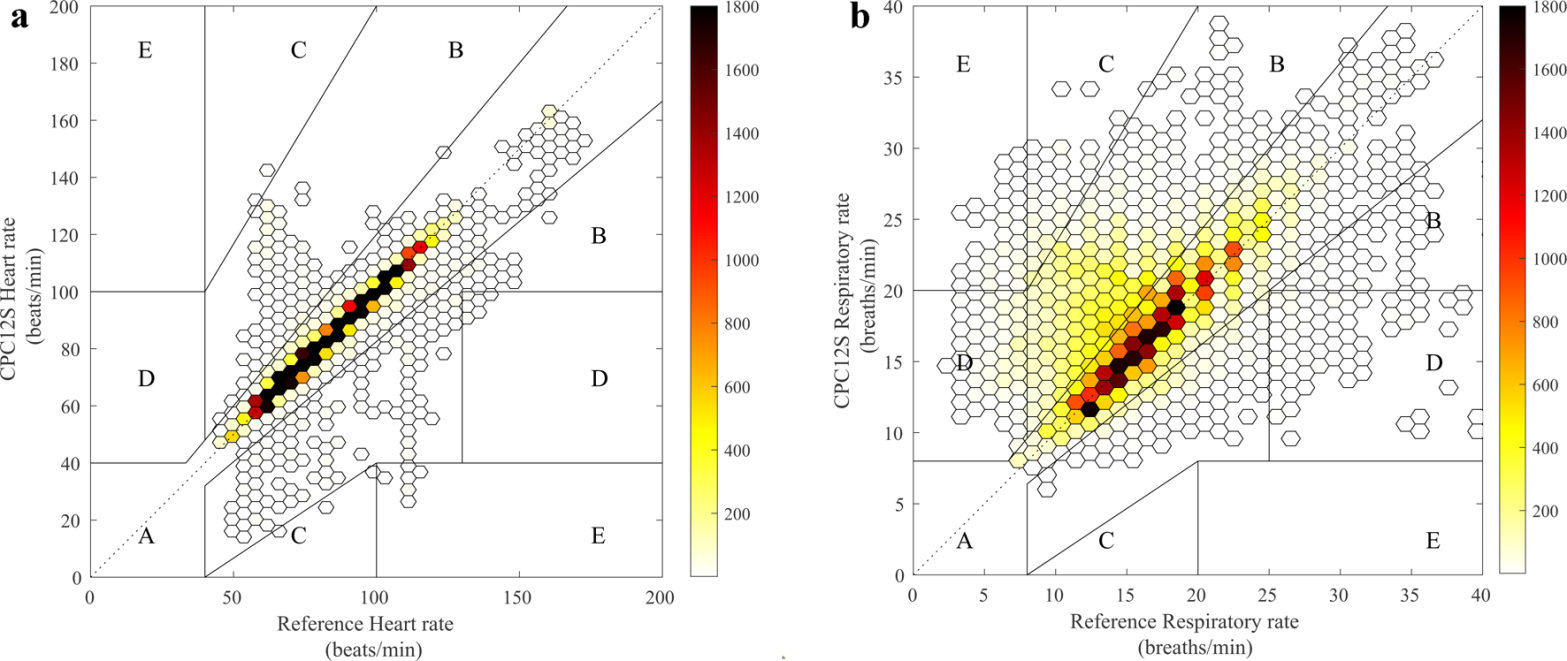
**(a and b)** Clarke Error Grid analysis to quantify clinical accuracy of heart rate measurements (**a**; left panel) and respiratory rate measurements (**b**; right panel) with the CPC12S system compared with the reference standard. The colored dots are measurement pairs superimposed on the error grid boundaries, where the color intensity is proportional to the number of observations. Region A shows points within 20% of the reference monitor; region B contains points outside 20% of the reference, but not leading to unnecessary treatment. Region C contains points leading to unnecessary treatment, region D indicates a potentially dangerous failure to detect e.g., bradycardia or tachycardia, and region E represents points where events are confused (e.g., bradycardia with tachycardia) in case of heart rate measurements

In total, 68,148 h measurement pairs were available for analyses in 70 patients. The overall bias was 0.0 bpm with narrow LoA of -3.5 to 3.4 bpm, indicating high accuracy and precision (Fig. 2a; Table 2). These results were within the predefined acceptable range. Supplementary file 1 (Fig. 6a-d) show Bland-Altman plots of subanalyses in each center. Figure 3a illustrates the Clarke Error Grid analysis with data pairs from all centers and Table 3 includes the percentage of data pairs in region A to E for each reference standard and the pooled results from all reference standards. Overall, adequate treatment decisions (zone A or B) would have been made in 99.2% with the CPC12S system. No measurements from Stockholm or Leuven, and few (0.9% or less) from Utrecht and Aachen were within regions C, D, or E, suggesting that very few HR measurements would result in failure to treat, unnecessary treatment or confusion between bradycardia and tachycardia.

### Respiratory rate

A total of 61,341 RR measurement pairs were available for analysis in 67 patients. RR-data from the reference standard was missing in three patients. Bias (mean difference) from the measurements in Utrecht, Stockholm and Aachen was within the predefined accepted range (Table 2). Results from a small group of patients in Leuven (*n* = 3) overestimated RR, with a bias of 4.1. Measurements from Stockholm and Aachen had the narrowest LoA for RR (Table 2). The pooled results indicate slight RR overestimation, with a bias of 1.5 brpm within the predefined range and LoA of -3.7 to 7.5 brpm. Figure 2b and Supplementary file 2 (Fig. 7a-d) show Bland-Altman plots of all subanalyses. Table 3; Fig. 3b show Clarke Error Grid analyses of the pooled RR-measurements. Overall, adequate treatment decisions (zone A or B) would have been made in 92.0% of all RR measurements (Table 3). Figure 3b; Table 3 show that 16.9% of the Leuven (*n* = 3) comparisons were within region C, indicating potential unnecessary treatment. In comparison, only 0.2% of the Stockholm measurements were were within region C, 1.4% in region D, and 0% in region E, implying that very few readings would lead to failure to treat, unnecessary treatment or confusion between bradypnoea and tachypnoea.

**Table 2:** Bland-Altman analysis of the CPC12S^a^ system versus the reference monitor in each hospital

	Number of measurement pairs	Number of patients	Bias	Lower 95% LoA^b^	Upper 95% LoA^b^	Lower 95% MEM^c^	Upper 95% MEM^c^
*Heart Rate*							
CPC12S - Reference Utrecht	33,909	18	0.5	-14.5	15.5	-6.7	7.8
CPC12S - Reference Stockholm	29,559	25	0.3	-5.2	5.7	-2.1	2.7
CPC12S - Reference Aachen*	3,034	24	-0.8	-9.9	8.3	-5.1	3.5
CPC12S - Reference Leuven	1,646	3	-0.1	-1.2	1.1	-0.8	0.7
CPC12S - Pooled analysis	68,148	70	0.0	-6.5	7.1	-3.5	3.4
*Respiratory Rate*							
CPC12S - Reference Utrecht	28,692	18	2.8	-6.5	12.1	-3.9	9.5
CPC12S - Reference Stockholm	29,068	25	1.2	-4.0	6.3	-4.2	6.6
CPC12S - Reference Aachen*	2,626	21	1.2	-5.8	8.1	-2.6	4.9
CPC12S - Reference Leuven	955	3	4.1	-4.3	12.5	-0.3	8.5
CPC12S - Pooled analysis	61,341	67	1.5	-6.0	8.9	-3.7	7.5
*Oxygen saturation*							
CPC12S - Reference Utrecht	22,993	16	1.6	-3.5	6.6	-1.8	5.0
CPC12S - Reference Stockholm	24,661	23	-1.0	-7.0	5.2	-4.6	2.7
CPC12S - Reference Aachen*	2,289	24	0.9	-4.6	6.3	-2.9	4.6
CPC12S - Reference Leuven	1,255	3	0.0	-5.0	4.9	-3.9	3.9
CPC12S - Pooled analysis	51,198	66	0.4	-6.7	7.6	-3.1	4.0
*Mean Arterial Pressure*							
CPC12S - Reference Utrecht	27,774	15	1.3	-31.9	34.6	-21.3	23.9
CPC12S - Reference Stockholm	23,217	22	9.2	-26.0	44.5	-16.8	35.3
CPC12S - Reference Aachen*	2,338	24	2.6	-32.2	37.3	-27.1	32.3
CPC12S - Reference Leuven	1,991	3	40.1	14.8	65.5	6.9	73.3
CPC12S - Pooled analysis	55,320	64	8.9	-30.3	54.0	-23.3	41.2
*Temperature*							
CPC12S - Reference Utrecht	13,925	11	-1.0	-2.7	0.7	-2.3	0.3
CPC12S - Reference Stockholm	1,026	2	-0.4	NaN	NaN	NaN	NaN
CPC12S - Reference Aachen*	2,505	21	-1.0	-2.6	0.6	-2.1	0.2
CPC12S - Reference Leuven	1,874	2	-1.3	NaN	NaN	NaN	NaN
CPC12S - Pooled analysis	19,330	36	-0.6	-1.8	1.2	-1.7	0.6

*3-lead ECG CPC system. ^a^CPC12S = Checkpoint Cardio 12 S system; ^b^LoA=Limits of Agreement; ^c^MEM= mixed effects model; ^d^NaN = Not a Number

### Oxygen saturation

**Fig. 4:**
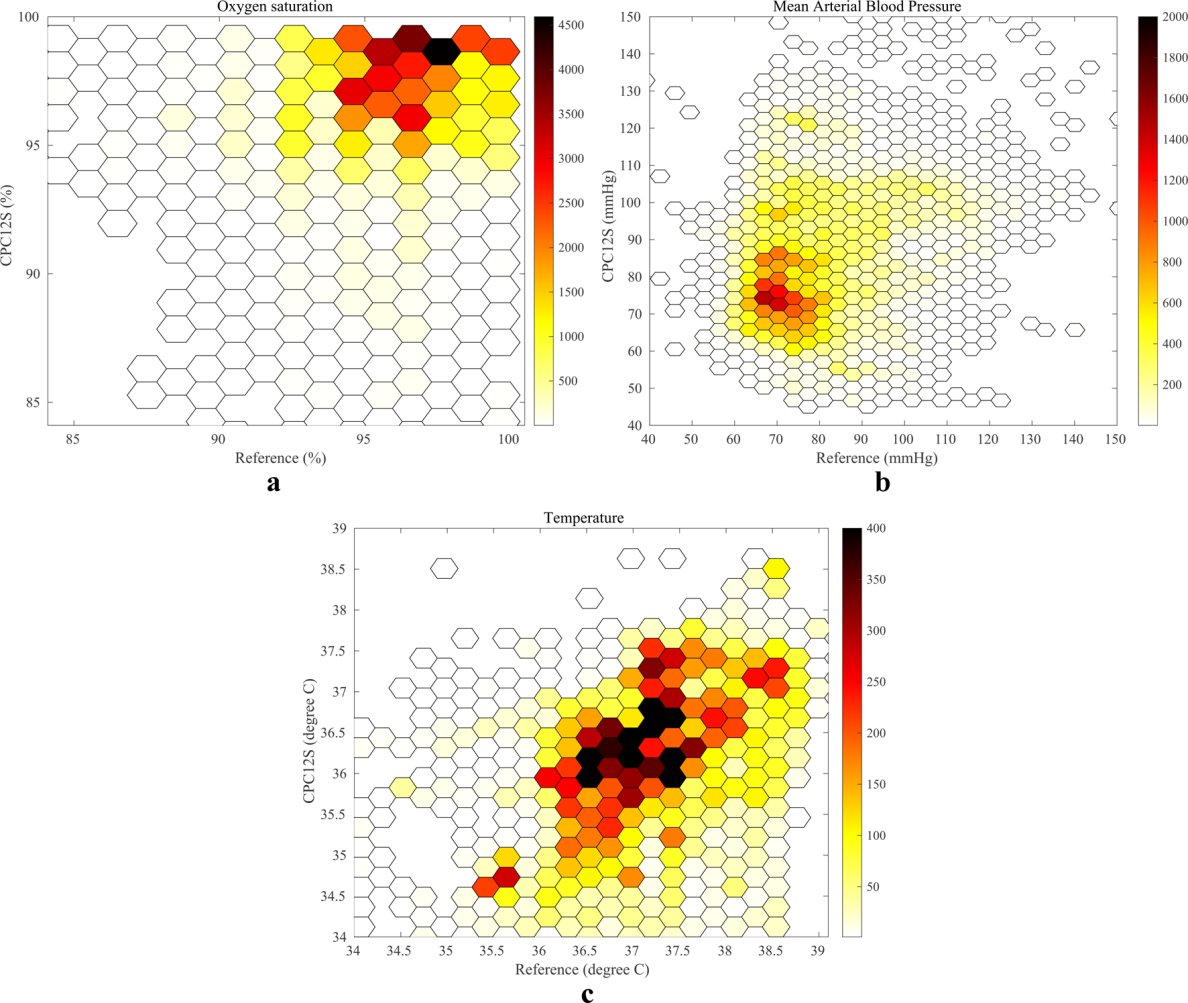
**(a)** Scatterplot comparing measurements of the pooled analysis of oxygen saturation with few (white) to many (dark red) measurement pairs from the CPC12S system and reference systems. (**b)** Scatterplot comparing measurements of the pooled analysis of mean arterial pressure with few (white) to many (dark red) measurement pairs from the CPC12S system and reference systems. (**c)** Scatterplot comparing measurements of the pooled analysis of temperature with few (white) to many (dark red) measurement pairs from the CPC12S system and reference systems

SpO2 data from the CPC12S system was missing in four patients and from the reference standards in three. In the remaining 66 patients, 51,198 SpO2 measurement pairs were available for analysis. The pooled analysis showed accurate results with a bias of 0.4% within the predefined range, but the LoA (-3.1–4.0%) was outside this range (Table 2; Fig. 2c). In Stockholm (*n* = 23), the CPC12S system showed a negative bias, with lower SpO2 readings than the reference standard, whereas Utrecht (*n* = 16) and Aachen (*n* = 24) CPC12S slightly overestimated SpO2 readings. Supplementary file 3 (Fig. 8a-d) illustrate Bland-Altman plots of comparisons from each center. Figure 4a illustrates a scatterplot of all SpO2 readings with few SpO2 readings below 95%.

### Blood pressure

In total, 55,320 measurement pairs of MAP were available for analysis in 64 patients. All MAP analyses showed wide LoA (Table 2). Subanalyses from Utrecht (*n* = 15) and Aachen (*n* = 24) indicated an acceptable bias of 1.3 mmHg and 2.6 mmHg respectively, but very low precision with wide LoA varying from − 27.1 mmHg to 32.3 mmHg. Data from Leuven (*n* = 3) showed that the CPC12S system greatly overestimated MAP with a bias of 40.1 mmHg. The pooled analysis indicated overestimation of MAP with a bias of 8.9 mmHg and wide LoA of -23.3 to 41.2 mmHg (Fig. 2d; Table 2). Supplementary file 4 (Fig. 9a-d) illustrate Bland-Altman plots of data from each center. A scatterplot of the BP readings (Fig. 4b) illustrates large variation with both under- and overestimation, most pronounced when MAP > 80 mmHg.

### Temperature

Temperature data from the reference standard was only available in 36 patients, resulting in 19,330 measurement pairs. Overall, the mean difference showed slight underestimation, with a bias of -0.6°C and LoA of -1.7 to 0.6 °C (Fig. 2e; Table 2). Supplementary file 5 (Fig. 10a-d) illustrate Bland-Altman plots of each subanalyses. Figure 4c shows a scatterplot of all temperature readings with the majority of measurements showing a small difference.

**Table 3:** Clarke error grid analysis to quantify clinical accuracy of all vital signs

	Zone A	Zone B	Zone C	Zone D	Zone E	Zone A + B
	*n* (%)	*n* (%)	*n* (%)	*n* (%)	*n* (%)	*n* (%)
**Heart Rate**						
CPC12S - Reference system Utrecht	87.8	11.0	0.2	0.9	0.1	98.8
CPC12S - Reference system Stockholm	91.4	8.6	0.0	0.0	0.0	100
CPC12S - Reference system Aachen*	98.7	1.0	0.0	0.3	0.0	99.7
CPC12S - Reference system Leuven	100	0.0	0.0	0.0	0.0	100
CPC12S - Pooled analysis	98.2	1.0	0.7	0.1	0.0	99.2
**Respiratory Rate**						
CPC12S - Reference system Utrecht	49.4	41.3	2.7	5.4	1.2	90.7
CPC12S - Reference system Stockholm	76.4	22.0	0.2	1.4	0.0	98.4
CPC12S - Reference system Aachen*	76.0	19.9	2.5	1.2	0.4	95.9
CPC12S - Reference system Leuven	53.0	26.2	16.8	2.0	2.0	79.2
CPC12S - Pooled analysis	69.1	22.9	5.0	2.1	0.9	92.0

*3-lead ECG CPC12S system. ^a^CPC12S = Checkpoint Cardio 12 S system

### Example of a patient measurement

**Fig. 5:**
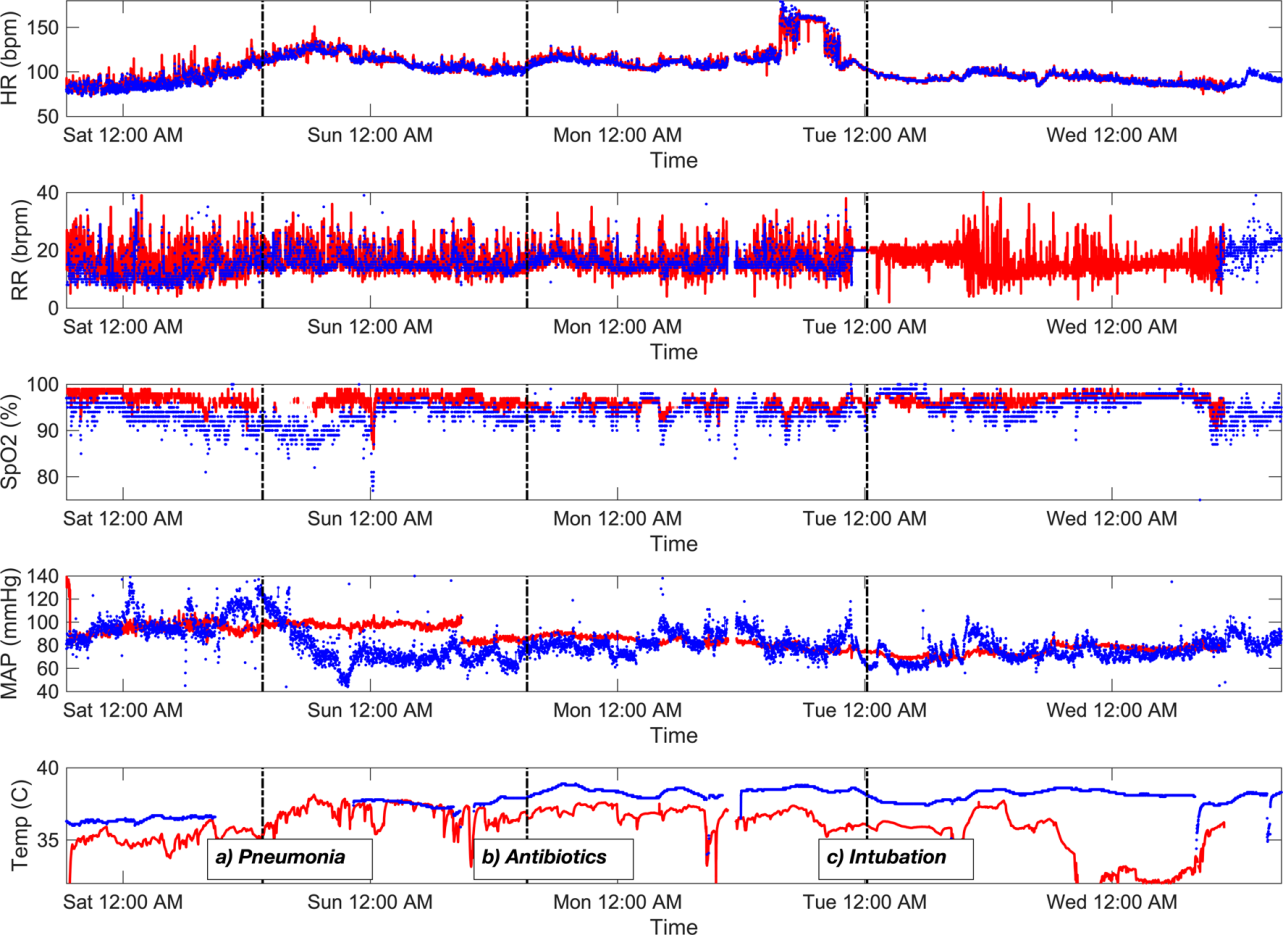
Example of a patient that is being continuously monitored for more than four days with the CPC12S system (red) and reference standard UMC Utrecht (blue). From top to bottom, the panels show heart rate (HR), respiratory rate (RR), oxygen saturation (SpO2), mean arterial pressure (MAP) and temperature measurements. Three clinical events occurring are marked in the lowest panel. This example shows unfiltered data from both systems

In Fig. 5, a patient’s vital signs measured with both the CPC12S system and a reference standard during the first four postoperative days in the ICU are illustrated. Three important clinical events occurred during this period; (a) a gradual increase in HR, decrease in SpO2 and subfebrile temperature occurred and the patient was diagnosed with pneumonia, (b) therapy was initiated with high-flow oxygen and antibiotics administered. Respiratory insufficiency led to intubation at the time of (c). New onset atrial fibrillation occurred approximately eight hours before the intubation and can be seen as a sudden increase in HR. This example illustrates agreement between HR and RR measurements recorded with the CPC12S system and the wired reference standard. Note that RR derived from the CPC12S shows more variation compared to the reference standard, while maintaining agreement. SpO2 readings of the CPC12S system overestimates SpO2 most pronounced until the second postoperative day at midnight. Blood pressure from the CPC12S system is not in agreement with those from the reference monitor. Axillary temperature measurements from the CPC12S system underestimate core temperature in comparison to the reference standard. Trends of increasing temperature are tracked, but numerous drops in temperature occur, especially during the last two days. A second example of a patient that is being continuously monitored is shown in Supplementary file 6 (Fig. 11).

## Discussion

In this multicenter method comparison study, we analyzed a novel multi-parameter wearable sensor designed to continuously monitor the full range of vital signs in patients after major non-cardiac surgery. Results show that the CPC12S system can accurately measure HR with high precision. Respiratory rate was slightly overestimated, with bias within the predefined accepted range. Overall, SpO2 readings were slightly overestimated, but this varied between reference systems. The CPC12S system with the axillary sensor underestimated reference core temperature measurements, and the readings showed frequent transient temperature drops resulting from poor sensor-skin contact during patient movement. Overall, accuracy of RR, SpO2 and temperature measurements were considered acceptable to track trends, but the LoA were outside the predefined accepted range. In contrast, blood pressure measurements showed low accuracy against all reference systems, suggesting that the current PTT-based method of continuous BP measurement is not accurate enough to be used clinically.

The present study shows that for HR, the CPC12S system provides similar monitoring agreement to wired reference standards. Few other clinical validation studies show similarly high levels of accuracy for chest-based wireless sensors that derive HR from ECG [23, 31]. HR derived from ECG outperforms wearable sensors using photoplethysmography or ballistocardiography to derive HR, especially during episodes of atrial fibrillation [23]. Even though the CPC12S system slightly overestimated RR, robust readings to track trends in patients’ physiology were obtained. Previous wearable device studies show much wider variation in measurements of RR, implying that reliable RR readings are more difficult to acquire than HR [23, 31, 32]. However, it must be noted that, despite its common use in ICU-grade monitors, thoracic impedance RR measurement cannot be considered a gold standard– as it is influenced by factors other than respiration, in particular patient movement. Consequently, RR showed more variation in patients who were moving or talking, and hence an unknown part of the measurement error can be attributed to inherent limitations of reference standards.

Since 80% of desaturation episodes are missed with intermittent spot checks on general wards [7], reliable continuous SpO2-monitoring outside high-care facilities is highly desirable. However, little is known about the accuracy of medical-grade wireless sensors for continuous SpO2 monitoring, since clinical validation studies are limited. A recent study compared vital parameters obtained during heart catheterizations by means of a photoplethysmography-based wristband (CardioWatch, Corsano Health, the Netherlands) with a pulse oximeter finger clip and reported accurately obtained SpO2 values with a bias of 0.54% nd 95% oA from − 3.1% o + 4.0%.These results cannot be compared to the results of SpO2 accuracy from the present study since these measurements were obtained under ‘controlled’ conditions of heart catheterizations during a short period of time when patients barely move [33]. Another recent study assessed the reliability of the wearable Radius PPG system (Masimo, Irvine, CA, USA) in recovering trauma patients during a 30-min period at the PACU and reported a clinically acceptable bias of 0.4%, simlar to our study findings, but with 95% LoA f -2.3% to + 0.1% outsde the clinically acceptable range [34]. Most other studies have investigated the performance of consumer-grade pulse oximeters, but these devices are designed for manual ‘spot-checks’ rather than continuous measurements [35–37]. As such, findings cannot be translated to validate the continuous performance of SpO2 monitoring with wearable devices. One study in 973 patients comparing SpO2 measurements from a smartwatch (Apple Watch [Apple Inc, Cupertino, California]) to medical-grade pulse oximeters, reported 95% LoA varying fro − 5.8% to + 5.9% [38]. OurSpO2 resuts show narrower LoA and did not exclude measurements during patient movement, whereas SpO2 readings from the Apple Watch were only obtained within time windows without motion [38, 39]. Other studies assessing validity of medical-grade wearable sensors for continuous SpO2 measurements were all obtained in healthy volunteers in controlled laboratory settings [38, 40]. A validation study during daily activities in healthy volunteers with the medical-grade sensor that measures SpO2 from an upper arm PPG sensor (Everion [Biovotion AG, Zürich, Switzerland]) showed an underestimation of SpO2 of > -1.1%, with LoA from − 4.6% to 2.5% [38].

The accuracy of BP measurements from the CPC12S system using PTT to derive BP was unacceptably low in comparison to invasive arterial line measurements. Accurate measurements are dependent on the presence of both valid PPG and ECG waveforms to calculate PTT and derive BP. Noise in the PPG signal due to motion artefacts or other reasons for signal distortion disturb the PTT calculation and consequently BP estimation. Secondly, vasoactive drugs administered to some of the study patients can cause vasoconstriction or vasodilatation that changes the peripheral resistance of the vessel walls resulting in an apparent decrease or increase of the PTT. Third, even though intra-arterial catheters for invasive BP measurements are considered gold standard, patient movement, blood clots or air bubbles may result in inaccurate BP readings in several patients [41]. In addition, an overdamped or underdamped arterial pressure waveform, not corrected in time by clinicians, may have occurred which could result in inaccurate BP readings as well. Furthermore, it is known that non-invasive BP with an oscillometric monitor and invasive BP measurements recorded at the same time can be discordant [42]. For these reasons, an unknown part of the observed difference in BP might be related to inaccurate invasive BP readings from the reference standard rather than the CPC12S system. However, the prototype version of this sensor system also showed poor agreement with measurements of a non-invasive blood pressure cuff in a previous study with volunteers executing a test protocol [18], which is in line with results from the present study. For these reasons, we suggest a different clinical validation approach for such measurements in future studies. First, measurements with continuous invasive BP from the reference monitor and non-invasive BP measurements from the wearable sensor should be restricted to periods of minimal or no patient motion, which might be achieved by using an accelerometer often present in wearable sensors. Secondly, an artificial intelligent algorithm can be used to confirm the presence of both a valid ECG, a valid PPG waveform and to verify sufficient quality of the invasive arterial blood pressure. At least in theory, agreement between BP from the wearable sensor system and an arterial line reference BP might improve. Finally, it is conceivable– even with these modifications - that valid PPG-based continuous blood pressure measurement will only be possible in the absence of vasoactive drug infusion, or after recalibration of the wearable sensor system. Nonetheless, other wearable sensor systems exist that use PTT to derive BP. According to the manufacturer, the accuracy of the ViSi Mobile system (Sotera Wireless, Inc. San Diego, CA, USA) consists of a mean error less than ± 5 mm Hg with a SD of ≤ 8 mm Hg [43]. However, these results were obtained in volunteers, under controlled laboratory conditions, and consists of few measurements recorded with cuff based BP [44]. The Biobeat (Biobeat Technologies, Petah Tikva, Israel) wristband showed a mean bias of -1.9 mmHg for systolic BP, but these results were evaluated with a cuff-based 24 h ambulatory BP monitor during daily activities in volunteers [45]. These findings can not be translated to patients at risk of clinical deterioration.

Temperature measured by CPC12S was underestimated in comparison to the reference standard. This could partly be explained by the difference of measuring temperature with a thermistor in the axilla to core temperature with a urinary bladder catheter. The CPC12S was, however, capable of detecting trends of increasing temperature. Transient episodes of apparent low temperature (< 34 $\:^\circ\:$C) are unlikely to reflect body temperature, but could result from sensor malposition (loss of skin contact resulting in sensor exposure to room temperature). In addition, the temperature sensor needs time to warm up to axillary temperature after (re)placement. Future algorithms may be designed to automatically recognize and ignore these transient temperature sensor dislocations. Since this has not been corrected for in the present study, it could explain part of the underestimation. To our knowledge, no previous study on continuous wireless monitoring of temperature in hospitalized patients exists. One study validated the accuracy of a wearable patch sensor (SensiumVitals [Sensium Healthcare Ltd., United Kingdom]) measuring axillary temperature in postsurgical patients and found low correlation with manually recorded tympanic measurements from the hospital’s electronic patient record (EHR) form [46]. However, when used by nurses as part of routine patient care, this method is inherently flawed, since time-stamped EHR data seldom corresponds with the actual time of manual measurements.

Even though the accuracy of the CPC12S system was not within the predefined standard for all vital parameters, the clinical utility is potentially high. Using a system like this in low-care wards may inform clinicians in time to improve the management of several adverse events which are common in the postoperative setting, such as arrythmias, fever as a possible early indicator of sepsis or respiratory insufficiency.

However, we need to highlight the different settings in which the results of the present study were obtained. The predefined accuracy boundaries chosen in this study may be considered wide during controlled conditions, but not during unsupervised monitoring of patients in general wards where more variation occurs during periods of e.g., movement. Accuracy specifications from bench tests of ICU-monitoring systems are usually obtained under ‘ideal’ conditions where comparison of beat-by-beat measurements may show perfect concordance, but does not reflect real-life clinical performance. In clinical practice, ICU staff looking at the bedside monitor ‘filter’ signal disturbances or artefacts when interpreting the patient’s condition. In addition, it is known that reliable measurements of RR, SpO2 and BP are difficult to acquire during periods of patient movement. Moreover, the intended use of these new wireless and wearable sensor systems is continuous monitoring to detect deteriorating vital sign patterns *over time*. This differs from the monitoring of critical patients in the ICU where deterioration of vital parameters has to be detected *instantly*. It is therefore unreasonable to expect continuous, wearable monitoring systems to have similar restrictive limits of agreement as those obtained under controlled conditions. No guidelines exist for acceptable LoA with continuous vital signs monitoring devices in clinical practice i.e., including mobilized patients as well as deteriorating patients with aggravating vital signs. It is therefore desirable to define new acceptable accuracy limits accounting for real-life clinical performance settings as well as vital sign values in the abnormal physiological range. In addition, future studies should focus on the ability to reliably detect trend patterns over time, as opposed to beat-by-beat accuracy.

The potential added value of continuous remote wireless patient monitoring for care processes, patient outcomes and resource utilization is increasingly recognized [47–50]. A recent ‘before and after’ comparison study introducing a continuous vital signs monitoring system in 4,769 medical and surgical patients, with historical controls, reported a reduction of one-third unplanned ICU admissions and rapid response team calls in the intervention group [51]. Studies in patients on surgical wards report a reduction in ICU admissions [15] and significant reduction of complications [52] when continuous monitoring of vital signs was used compared to intermittent spot checks. Klik of tik om tekst in te voeren. However, current evidence of wearable wireless continuous monitoring devices on clinical outcomes is still sparse, since most studies are inherently limited by their retrospective, before and after approach or are underpowered to demonstrate significant impact on patient outcomes [53]. Therefore, large prospective trials are necessary to obtain evidence of the impact of continuous vital signs monitoring on patient outcomes.

## Limitations

This current study has several limitations. The number of patients studied, the observation time available for agreement analysis and the sampling rate of vital signs from the reference systems varied among the four study centers. In addition, no data on weight and BMI were collected and as such it is unknown whether a high BMI may affect accuracy of the obtained parameters. However, a previous validation study did not show different results in volunteers with a high BMI [18]. Additionally, no separate analysis was performed excluding atrial fibrillation episodes, which could affect agreement of BP and SpO2. Furthermore, in half of the patients, continuous temperature was not measured with the reference standard. However, given the large amount of monitored time and measurement pairs available, we believe valid conclusions can be drawn regarding the reliability of the CPC12S monitoring system for each of the vital signs measured. Another limitation is the fact that the time to intervene is quicker in HDUs/ICUs as compared to low-care ward settings due to the higher nurse-to-patient ratio. Therefore, the duration of measurement pairs with abnormal physiological values - such as a critically low RR (< 8 brpm) or periods of hypoxemia (SpO2 < 90%) is likely limited. Consequently, validating continuous wearable monitoring systems in abnormal physiological ranges in high-risk settings remains difficult.

No previous study exists comparing an ‘all-in-one’ vital sign monitoring solution, capable of continuously measuring HR, RR, SpO2, BP and temperature, to several reference monitoring systems in different hospital environments. In our study, a large amount of measurement pairs was available for analysis. This study demonstrates a high variability in measurement performance of CPC12S and the reference systems. Subanalyses from Stockholm and Aachen show similar results, which could be explained by the fact that both hospitals use an ICU monitoring system from the same manufacturer. Therefore, an unknown part of the observed measurement error might be related to variable accuracy levels of the specific ICU monitor used as reference standard rather than the wearable monitoring system studied [54]. This emphasizes the importance of testing new wearable sensors against several reference systems in clinical practice.

## Conclusion

The tested CPC12S multiparameter system accurately measures HR, in comparison to wired reference standards. The accuracy of RR and SpO2 readings were slightly overestimated, with LoA outside the predefined clinical acceptable limits. Axillary temperature measurements underestimated core temperature and showed occasional transient drops in readings with LoA outside the predefined acceptable limits, but trends of increasing temperature were tracked and could be useful in early detection of fever. The accuracy of BP measurements was unacceptably low, and must be improved. The novel approach of comparing a wireless monitoring system to several different clinically used reference systems provides valuable insights to the performance of such systems and could become a useful approach for validation. It should be noted that although accuracy of such systems is essential, usability, acceptability and costs are important factors for consideration before implementation in clinical practice.

## Electronic supplementary material

Below is the link to the electronic supplementary material.

Supplementary Material 1

Supplementary Material 2

Supplementary Material 3

Supplementary Material 4

Supplementary Material 5

Supplementary Material 6

## Data Availability

No datasets were generated or analysed during the current study.
