# Supplementary material for: Reliability of an all-in-one wearable sensor for continuous vital signs monitoring in high-risk patients: the NIGHTINGALE clinical validation study
Source: J Clin Monit Comput. 2025 Mar 18;39(5):1087–100. doi: 10.1007/s10877-025-01279-x (PMC12474673; doi:10.1007/s10877-025-01279-x)
Supplement: Supplementary file 5 — Supplementary Material 5 [file 10877_2025_1279_MOESM5_ESM.docx]

**
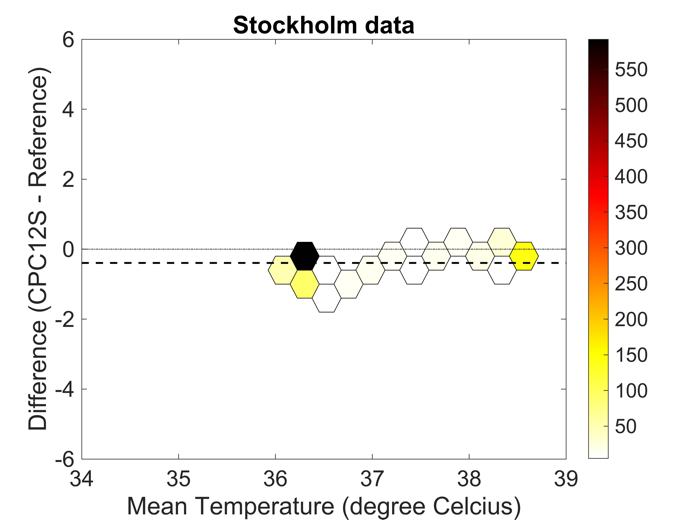
Supplementary file 5**


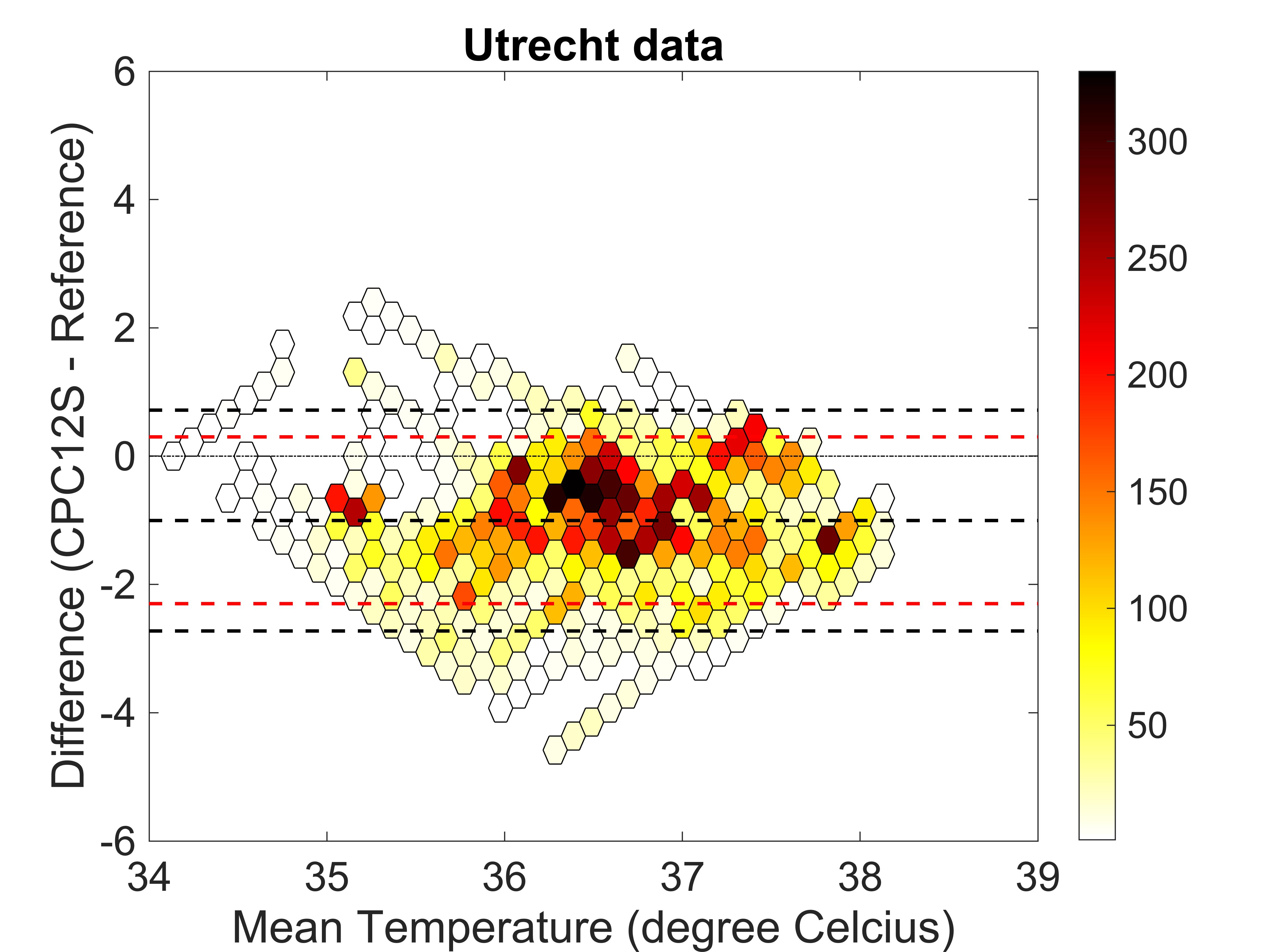


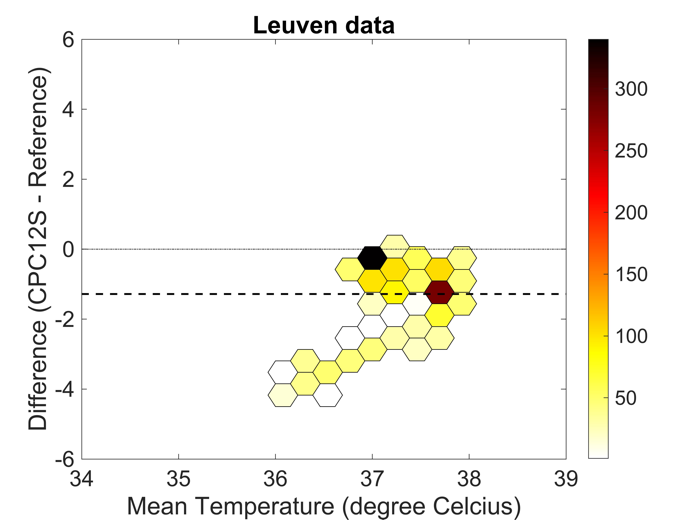

**Fig. 10a-d** Bland- Altman plots of temperature measurements for Utrecht, Stockholm, Aachen and Leuven respectively. Bias from the Bland-Altman method in black (if present), and limits of agreement from mixed effects models in red (if present).
