# Supplementary material for: Reliability of an all-in-one wearable sensor for continuous vital signs monitoring in high-risk patients: the NIGHTINGALE clinical validation study
Source: J Clin Monit Comput. 2025 Mar 18;39(5):1087–100. doi: 10.1007/s10877-025-01279-x (PMC12474673; doi:10.1007/s10877-025-01279-x)
Supplement: Supplementary file 6 — Supplementary Material 6 [file 10877_2025_1279_MOESM6_ESM.docx]

**Supplementary file 6**


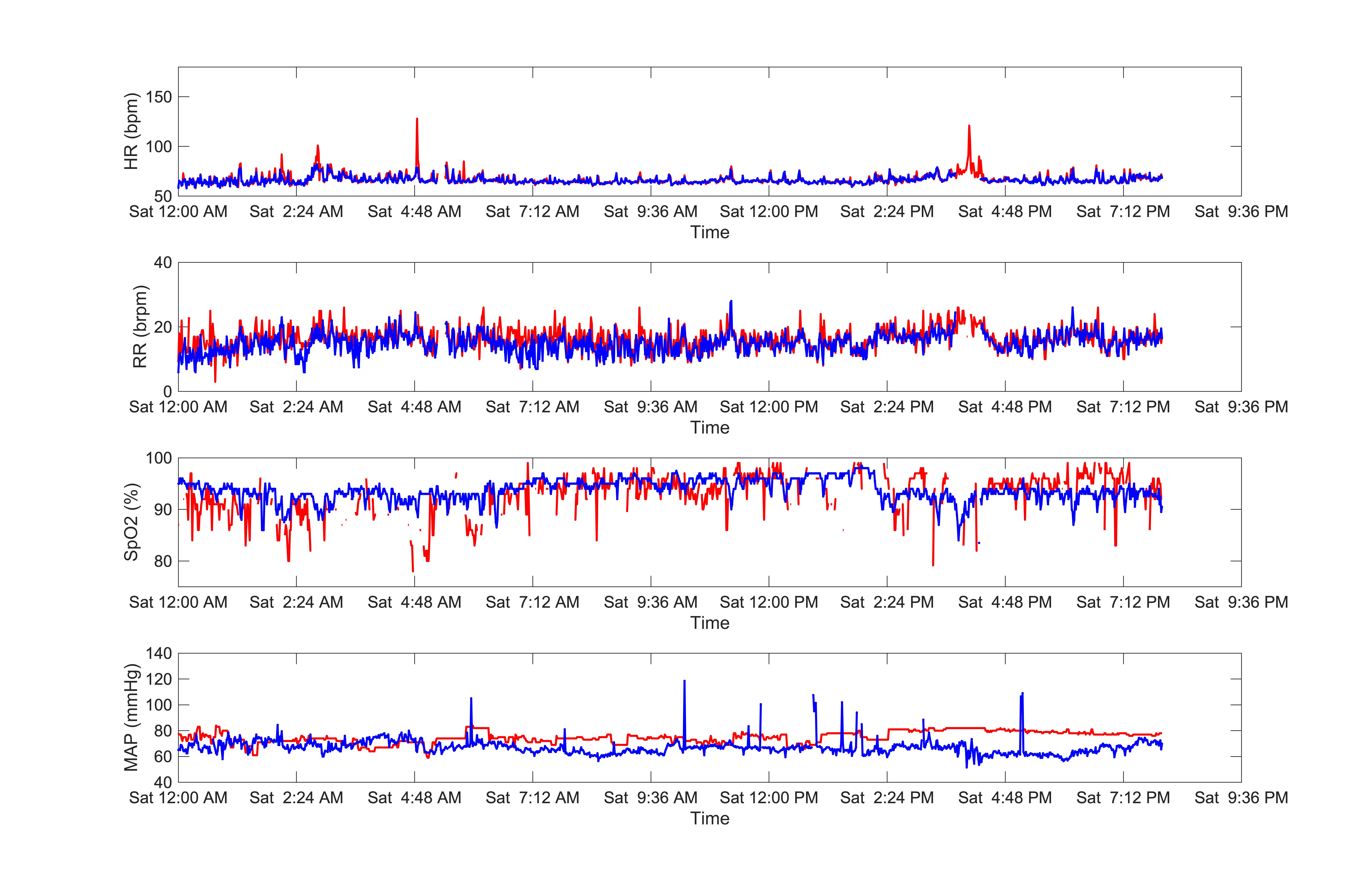


**Fig. 11** Example of a patient that is being continuously monitored with the CPC12S system (red) and reference standard Karolinska Hospital (blue). From top to bottom, the panels show heart rate (HR), respiratory rate (RR), oxygen saturation (SpO2) and mean arterial pressure (MAP). The patient did not have continuous monitoring of temperature with the reference system and therefore this vital sign is not displayed. This example shows unfiltered data from both systems.
